# Domestication and breeding objective did not shape the interpretation of physical and social cues in goats (*Capra hircus*)

**DOI:** 10.1038/s41598-023-46373-9

**Published:** 2023-11-04

**Authors:** Christian Nawroth, Katrina Wiesmann, Peter Schlup, Nina Keil, Jan Langbein

**Affiliations:** 1 Research Institute for Farm Animal Biology, Institute of Behavioural Physiology, 18196 Dummerstorf, Germany; 2 Swiss Federal Food Safety and Veterinary Office, Centre for Proper Housing of Ruminants and Pigs, Agroscope Tänikon, 8355 Ettenhausen, Switzerland; 3 Tierpark Bern, 3005 Bern, Switzerland

**Keywords:** Animal behaviour, Behavioural ecology, Animal breeding

## Abstract

Artificial selection by humans, either through domestication or subsequent selection for specific breeding objectives, drives changes in animal cognition and behaviour. However, most previous cognitive research comparing domestic and wild animals has focused on companion animals such as canids, limiting any general claims about the effects of artificial selection by humans. Using a cognitive test battery, we investigated the ability of wild goats (non-domestic, seven subjects), dwarf goats (domestic, not selected for milk production, 15 subjects) and dairy goats (domestic, selected for high milk yield, 18 subjects) to utilise physical and social cues in an object choice task. To increase the heterogeneity of our test samples, data for domestic goats were collected by two experimenters at two research stations (Agroscope; Research Institute for Farm Animal Biology). We did not find performance differences between the three groups in the cognitive test battery for either physical or social cues. This indicates that for a domestic non-companion animal species, domestication and selection for certain breeding objectives did not measurably shape the physical and cognitive skills of goats.

## Introduction

Artificial selection by humans, either in the form of domestication or via subsequent selection for specific traits such as production parameters, is an important factor driving changes in the phenotype of domestic animals. Domestic animals differ in their appearance, anatomy, and physiology from their wild counterparts^1–3^. Changes in neural development have also led to striking changes in behaviour, such as increased play behaviour and decreased emotional reactivity towards humans^4,5^. In particular, an improved capacity to interact and communicate with humans by domestic animals is considered a key outcome of how domestication shaped the domestic brain^6–8^. As domestic animals live in an anthropogenic environment where problem-solving abilities are less crucial to survival, it has also been speculated that domestic animals might show a decreased ability to interpret physical/causal cues compared to their wild counterparts^9,10^. While most research in this area has focused on model species such as canids, broader inferences on the impact of artificial selection by humans are only possible by testing a broader variety of domestic species, especially those with different selection backgrounds (i.e., domestic animals not selected for increased communication with humans).

Domestication has shaped how domestic species interact and communicate with humans in a variety of ways^4,11,12^. In dogs, domestication not only altered general behavioural patterns^13^, but it also led to an increased ability to interact and communicate with humans, such as following pointing gestures, interpreting human attentional states, and forming attachment bonds with humans^14–16^, but see^17^. These changes in their socio-cognitive abilities have been often ascribed to a reduction of emotional reactivity towards humans over the course of domestication^4,18^, with epigenetic and developmental effects being also discussed^8,19^. Experimental proof of this interconnection comes from an experimental population of silver foxes that showed similar enhanced socio-cognitive traits after a controlled artificial selection for reduced emotional reactivity towards humans^4^. In addition, heritability estimates have shown that the inclination to interact with and use information from humans, has a genetic basis in dogs^20,21^. Others have also stressed that next to domestication, the degree of socialization with humans during ontogenesis and the level of training by humans based on conditioning can have an impact on the understanding of human social cues by domestic animals^22,23^. Next to the interpretation of social cues, it has also been argued that domestication might have affected the interpretation of physical cues, and problem-solving abilities in general^10,17^. Humans do not only protect domestic animals from predators, but they often also provide food and shelter, so domestic animals do not have to rely heavily on dealing with these problems themselves. However, recent research on canids’ comprehension of their physical environment, e.g., their ability to track the movement of hidden objects or show means-end understanding in a support task, does not lend support to this hypothesis—wolves and dogs performed equally well in these tasks^24,25^.

Further selection for specific traits, and not just general domestication effects, might have an impact on the comprehension of physical and social cues. Working dog breeds have been shown to excel in their use of human-given cues to locate a reward compared to non-working dog breeds^26,27^. However, breeding objectives can vary depending on the domestic species at hand. For domestic farm animal species, these breeding objectives most often include production traits, such as milk yield or meat quality and quantity, rather than parameters that are associated with companionship and/or increased cooperation with humans (e.g., in the case of hunting or herding). In domestic farm animals, breeding for high performance may lead to investing more resources into production traits and less in other biological processes, i.e. resource allocation theory^28^, which could also affect their ability to comprehend physical and social cues. Additionally, as handlers want to manage docile animals, selection for high production might have been indirectly often accompanied with a decreased emotional reactivity towards humans, which might have also led to an improved comprehension of human social cues^4^. Further investigations on the impact of selection for production traits are needed to make inferences on whether specific, direct or indirect, selection effects on learning and cognition, as described in dogs, are also at play when further selecting domestic animals for specific breeding objectives.

Most research investigating the impact of artificial selection by humans on the interpretation of physical and social cues has focused on species that have been selected for enhanced interactions with humans, such as dogs and ferrets^7,10,29^, but see^30^ for a comparison of domestic pigs and wild boar. Domestic farm animal species can serve as additional, complementary, model organisms for investigating the effect of artificial selection by humans on cognitive functioning—they offer the advantage that (1) their domestication histories focus on production, and not companionship with human, and offer a possibility to disentangle effects of domestication and further selection for specific traits; (2) in contrast to dogs, who often come from single households with unknown ontogenetic and environmental backgrounds, farm animals are often group-housed, so ontogeny and housing conditions can be better standardised, and (3) several wild types of many domestic farm animals, such as wild boar and bezoar goats, are still available for comparative approaches. Goats have been domesticated approximately 10.000 years ago^31^ and are thus considered as one of the first domesticated livestock species (although domestication of dogs is speculated to have happened about 25.000 years ago^32^), offering a range of breeds with different breeding objectives focusing on production parameters (meat, dairy, wool). We know from previous research that they proficiently engage in, and solve, a variety of physico- and sociocognitive tasks^33–35^, and not only show potential for complex social interactions with conspecifics^36^, but also with humans^37,38^. This makes them a suitable model species for investigating effects of artificial selection by humans, both via domestication and also via the selection for production traits.

Cognitive test batteries, i.e., a set of tasks that are presented over a short period of time to make inferences about different cognitive capacities of a study population, have been shown to be a reliable tool in detecting between- and within-species differences^39,40^. Using such a test battery comprising a variety of physical and social cues, we investigated whether domestication and/or a specific breeding objective (i.e., selection for high milk yield) has affected the ability of goats to use these cues to locate a food reward. We tested wild goats, and two selection lines of goats: dwarf goats and dairy goats. The Nigerian Dwarf goat is commonly kept as pet and zoo animal in Europe and not selected for productivity traits. The only selection aim in the Dummerstorf population was to avoid inbreeding. For our dairy goat subjects, we used three of the most common high-producing dairy breeds in Switzerland and Germany (Saanen and Chamois coloured goats, Deutsche Edelziege). To increase the heterogeneity of our sample, data for domestic goats was collected by two alternating researchers at two research sites (Agroscope Tänikon in Ettenhausen, Switzerland, and the Research Institute for Farm Animal Biology in Dummerstorf, Germany)^41–43^.

If domestication has led to an improved interpretation of social cues in goats, we expect to see domestic goats (dwarf and dairy) outperforming wild goats in the social domain. In turn, if domestication has led to a decreased ability to interpret physical cues in goats, we expect to see wild goats outperforming domestic goats (dwarf and dairy) in the physical domain. If further selection for production traits decreased emotional reactivity, we would expect that dairy goats (selection for milk production) outperform dwarf goats (no selection for production traits) in their use of social cues. If breeds selected for higher production invest more resources into production traits and less in other biological processes, we expect that dwarf goats would outperform dairy goats in their ability to use physical cues.

## Methods

### Subjects, housing and general procedure

Seven wild goats (4 males, 3 females; mean ± SEM, Tierpark Bern: 812 ± 192 days old, at start of testing), 18 female Nigerian dwarf goats (Ettenhausen: 446 ± 1.1 days old; Dummerstorf: 464 ± 6.8 days old at start of testing, 9 subjects at each site) and 18 female dairy goats (Ettenhausen: 412 ± 4.4 days old, Dummerstorf: approximately 457 days old at start of testing, 9 subjects at each site) participated in the experiments. Wild goats were tested in October 2018 and August 2020. Domestic goats were tested in April-June 2017 (Ettenhausen) and May–August 2018 (Dummerstorf).

Wild goats were bred and housed at the Tierpark Bern in Switzerland. They were group-housed in a large enclosure (3679 m^2^), within a mixed group of about 18 subjects of both sexes and varying ages. Dwarf goats for both locations were bred in Germany, Dummerstorf. Dairy goats were bred at different Swiss farms (Ettenhausen: Saanen and Chamois coloured goats) and one large German farm (Dummerstorf: Deutsche Edelziege). Initially, dwarf and dairy goats were housed in one large group pen of 30 animals (per selection line) at each location. At the age of 7–8 months, these goats were then moved to six group-pens of 10 goats each (see ESM and^44^ for more housing details).

The number of wild goats participating in the study was determined by the number of subjects that approached the apparatus and were motivated to interact with the experimenter (see ESM). The number of domestic goats for the current study was logistically determined due to their assignment for a specific treatment for a subsequent study (see^45^); i.e., tested domestic goats were a randomly chosen sub-sample of three subjects per pen and were thus assigned to one treatment group for a study that investigated the impact of test experience on individuals' performance in subsequent conceptually different cognitive tests at both locations (Ettenhausen: n = 9 for dwarf and dairy goats each; Dummerstorf: n = 9 for dwarf and dairy goats each).

**Figure 1:**
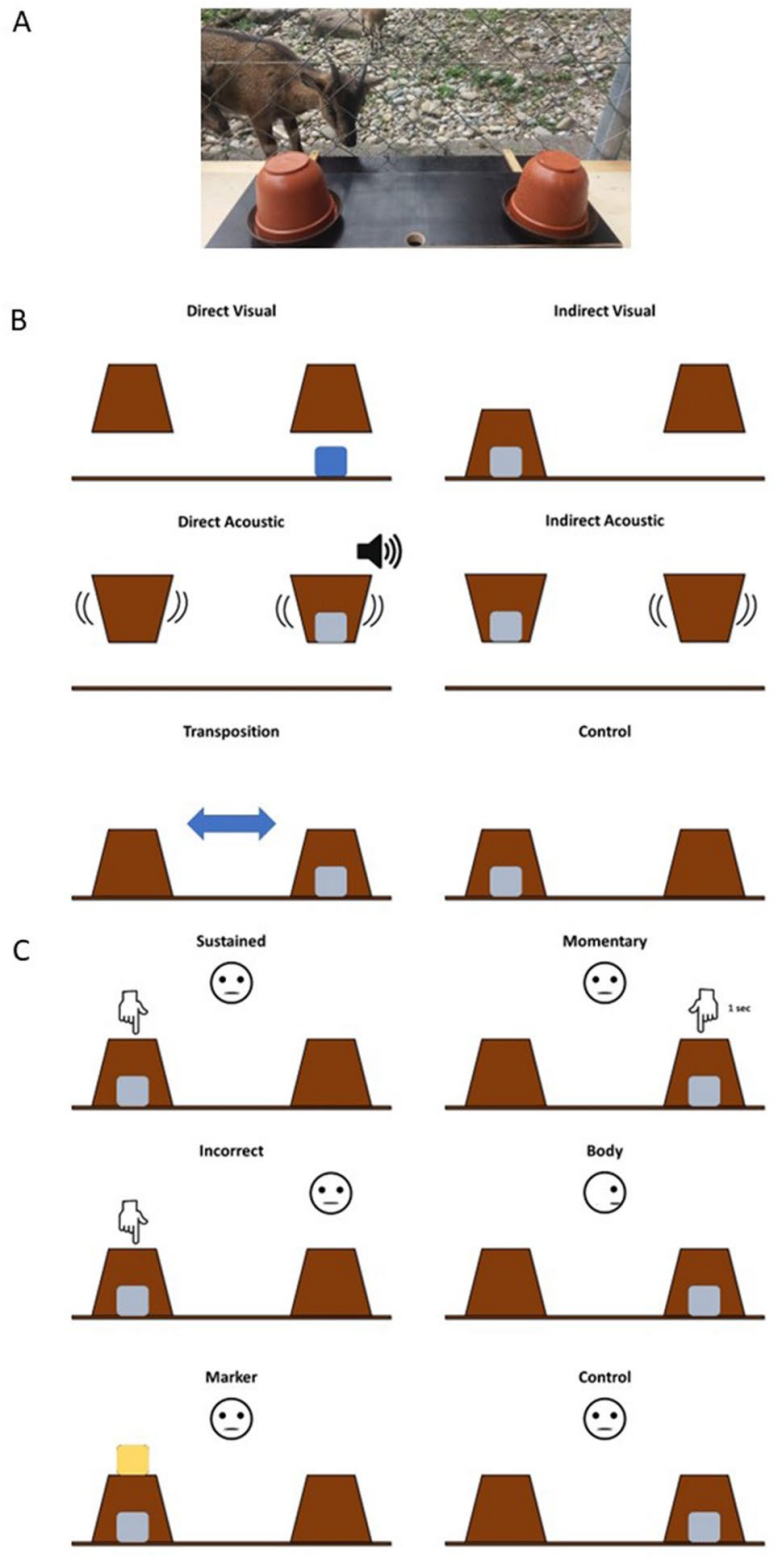
(**A**) setup of the test procedure when wild goats have been tested, including the sliding board and the two-choice options; (**B**) illustration of the five different physical cues, plus control condition; (**C**) illustration of the five different social cues, plus control condition.

For testing, wild goats remained in a group setting as it was not possible to separate individuals from the group (see ESM for habituation, shaping and training procedure). The experimenter sat outside of the enclosure separated from the tested animal by the enclosure fence. A sliding board (60 cm × 20 cm) was placed on a small table (105 cm × 40 cm) which was attached to the fence at a height of approximately 35 cm (see Fig. 1A). Goats could insert their snouts through the gaps in the fence to make a choice between objects presented on the sliding board. Subjects were not food restricted before testing. Wild goats were tested opportunistically whenever they were willing to participate in the test (~ between 9:00 and 12:00, and 13:00–16:00), so it was not possible to establish a certain testing order. The number of trials per day ranged from 1 to a maximum of 48 (i.e., 4 sessions, see below). Four of the subjects have been tested by CN, while three of the subjects have been tested by KW.

Domestic goats were physically and visually separated from their pen-mates in a designated test area (450 cm × 200 cm) next to a waiting area (600 cm × 200 cm) for testing. Habituation, shaping and training procedures for domestic goats are described in the ESM and in^44^. The experimenter sat in another adjacent compartment (150 cm × 200 cm) separated from the tested animal by a grate. A sliding board (60 cm × 20 cm) was placed on a small table (105 cm × 40 cm) at a height of approximately 35 (dwarf)/40 (dairy) cm in front of the grate. Goats were able to insert their snouts through the bars to make a choice regarding objects presented on the sliding board. Subjects were not food restricted before testing. Domestic goats were tested once a day (~ between 9:00 and 12:00, with time of testing counterbalanced between subjects). To decrease potential experimenter biases, two experimenters (CN and KW) were alternating in presenting the respective physical or social cues between each test session at both research sites.

### Cognitive test battery

In total, 40 goats were exposed to the test procedure (18 dairy goats, 15 dwarf goats, 7 wild goats). Three dwarf goats had to be excluded as they did not reach a respective habituation criterion (see ESM and^44^).

In the general experimental trial setup of the cognitive test battery, two cups of the same colour were positioned on the left and right side (brown; diameter 12 cm; height 10.5 cm) on top of bowls (brown; diameter 14 cm; height 2 cm) on the sliding table (Fig. 1a). Subjects received two motivation trials before each test session, where food was positioned in the uncovered bowls. In test trials, one cup was baited with a piece of dry pasta (domestic goats) or concentrate (wild goats) surreptitiously (except in the transposition condition, see below) and the experimenter administered five different physical or social cues to the subjects to indicate the correct position of the reward. In addition, control trials were included to control for any inadvertent cueing. In those, one cup was baited with a piece of dry pasta surreptitiously while the experimenter did not provide any information on the location of the reward.

All sessions containing physical cues were presented first to avoid interference of goats’ experience with social cues (e.g., due to local enhancement effects which might bias subsequent responses due to potentially learned contingencies) during the presentation of physical ones. The physical cues included (Fig. 1B):

- *Choice by visual exclusion (use of direct information)* The experimenter (E) touched and lifted both cups, providing the goats with direct visual information (‘presence of food’) regarding the location of the food reward.
- *Choice by visual exclusion (use of indirect information)* E touched both cups but lifted only the empty cup, providing the goats with indirect visual information (‘absence of food’) regarding the location of the food reward.
- *Choice by auditory exclusion (use of direct information)* E lifted and shook both cups, providing the goats with direct auditory information (rattling noise = ‘presence of food’) regarding the location of the food reward.
- *Choice by auditory exclusion (use of indirect information)* E lifted both cups but shook only the empty cup, providing the goats with indirect auditory information (no rattling noise = ‘absence of food’) regarding the location of the food reward.
- *Object permanence (transposition)* E baited one of the cups in full view of the subject. Next, E simultaneously moved the left cup to the right side and the right cup to the left side of the board so that the cups crossed paths in the middle. When crossing path, the baited cup was always moved towards the subject.
- *Control* E presented a baited and an unbaited cup and remained motionless until the subject has made a choice.

All physical test conditions including the control trials were presented in a pseudo-randomized order across sessions. To avoid olfactory cueing, a piece of pasta was attached inside each cup. All physical cues, except the transposition, were presented for approximately two seconds. Then, the experimenter moved the sliding board towards the grate and the subject could indicate a choice. For the transposition, the experimenter moved the sliding board towards the grate right after the movement of the cups. A session consisted of two trials per condition, totalling in 12 trials per session. Subjects received 12 sessions in total. Thus, each subject received 24 trials per condition. Each dairy and dwarf goat received one session per day, while wild goats could receive up to four sessions a day due to logistical limitations.

The social cues included (Fig. 1C):

- *Pointing (sustained)* E was positioned in the middle between both cups and pointed with his arm and finger at the baited cup (distance index finger-cup: approximately 3 cm). Their arm remained in this position until the subjects made a choice.
- *Pointing (momentary)* E was positioned in the middle between both cups and pointed with their arm and the finger at the baited cup for about 1 s (distance index finger-cup: approximately 3 cm).
- *Pointing (from incorrect position)* E was positioned behind the unbaited cup and pointed with their arm and finger at the baited cup (distance index finger-cup: approximately 3 cm).
- *Body* E was positioned in the middle between both cups and directed their body and head towards the baited cup. They remained in this position until the subject has made a choice.
- *Marker* E was positioned in the middle between both cups and placed a marker on top of the baited cup. The marker remained until the subject has made a choice.
- *Control* E presented a baited and an unbaited cup and remained motionless until the subject has made a choice.

All social test conditions including the control trials were presented in a pseudo-randomized order across sessions. To avoid olfactory cueing, a piece of pasta was attached inside each cup. All social cues were presented for approximately two seconds, except for the momentary pointing. Then, the experimenter moved the sliding board towards the grate and the subject could indicate a choice. A session consisted of two trials per condition, totalling in 12 trials per session. Subjects received 12 sessions in total. Thus, each subject received 24 trials per condition. Each dairy and dwarf goat received one session per day, while wild goats could receive up to four sessions a day due to logistical limitations.

If a subject did not indicate a choice after 30 s, the trial was repeated. If the subject did not make a choice in the repeated trial, the session was terminated. After three consecutive terminated sessions, a subject would have been excluded from further testing. None of the subjects had to be excluded. Due to the group testing of the wild goats, if another goat interfered in the trial of the focal goat, a trial would have been repeated once the focal goat approached the experimental apparatus again.

### Ethical note

Animal care and all experimental procedures were in accordance with the ASAB/ABS Guidelines for the Use of Animals in Research^46^. All procedures involving animal handling and treatment were approved by the Committee for Animal Use and Care of the Ministry of Agriculture, Environment and Consumer Protection of the federal state of Mecklenburg-Vorpommern, Germany (Ref. No. 7221.3-1.1-062/17) and by the Swiss Cantonal Veterinary Office, Thurgau (Approval No. TG04/17-29343). Housing facilities met the Swiss welfare requirements for farmed goats. All measurements were non-invasive, and the experiment lasted no more than 10 min for each individual goat per day. If the goats had become stressed, the test would have been stopped. The study is reported in accordance with ARRIVE guidelines (https://arriveguidelines.org).

### Data scoring and analysis

Digital video cameras (Ettenhausen and Bern: Sony HDR-CX240E; Dummerstorf: Panasonic HDC-SD60) were used to record all trials. We scored which cup (correct or incorrect) the test subject chose first for each trial. A ‘correct’ choice was scored if the subject chose the baited cup (i.e., by putting its snout through the respective gap in the grate).

To assess inter-observer reliability, 10% of the videos were coded by a second coder who was unfamiliar to the initial hypothesis. Inter-observer reliability for choice of a cup showed a high level of agreement (Cohen’s *κ* = 0.994, *P* < 0.0001).

Statistical analyses were carried out in R v.4.1.1^47^. The choice of goats in the trials was treated as a binary variable (correct = 1, incorrect = 0) and was analyzed with a generalized mixed-effects model (GLMM) fit with binomial family distribution and logit link function (GLMM; bglmer function, blme library^48^). Physical and social cues were analysed separately in one model each. Both ‘Condition’ (factor with six levels: physical cues: visual direct, visual indirect, acoustic direct, acoustic indirect, transposition, control; social cues: sustained, momentary, incorrect, body, marker, control) and ‘Group’ (factor with three levels: dairy, dwarf, wild) as well as their interaction were included as fixed factors. ‘Session’ (1–12) nested in ‘Identity’ of the goats nested in ‘Pen’ (1–12 for dwarf and dairy goats; 13 for wild goats) nested in ‘Location’ (Ettenhausen, Dummerstorf, Bern) was included as a random factor to control for repeated measurements. We also included ‘experimenter’ (CN and KW) as additional crossed random factor. For both models, we checked the residuals of the models graphically for normal distribution and homoscedasticity (simulateResiduals function, DHARMa library).

We followed a full model approach, i.e., we set up a maximum model that we present and interpret^49^. First, we calculated the global p-value (between the maximum and null model) using parametric bootstraps (1000 bootstrap samples, PBmodcomp function, pbkrtest library). If that model reached a low p-value, we tested each of the predictor variables (including their interaction) singly by comparing the full model to the one omitting this predictor. P-values calculated with parametric bootstrap tests give the fraction of simulated likelihood ratio test (LRT) statistic values that are larger or equal to the observed LRT value. This test is more adequate than the raw LRT because it does not rely on large-sample asymptotic analysis and correctly takes the random-effects structure into account^50^. Alpha level was set at 0.05 for both models.

Code and raw data are available at the Electronic Supplementary Material (ESM) and here: https://osf.io/fcyg3/.

## Results

The global model comparison yielded statistically supported differences in both models (physical cues: P = 0.0011; social cues: P = 0.0011).

**Figure 2:**
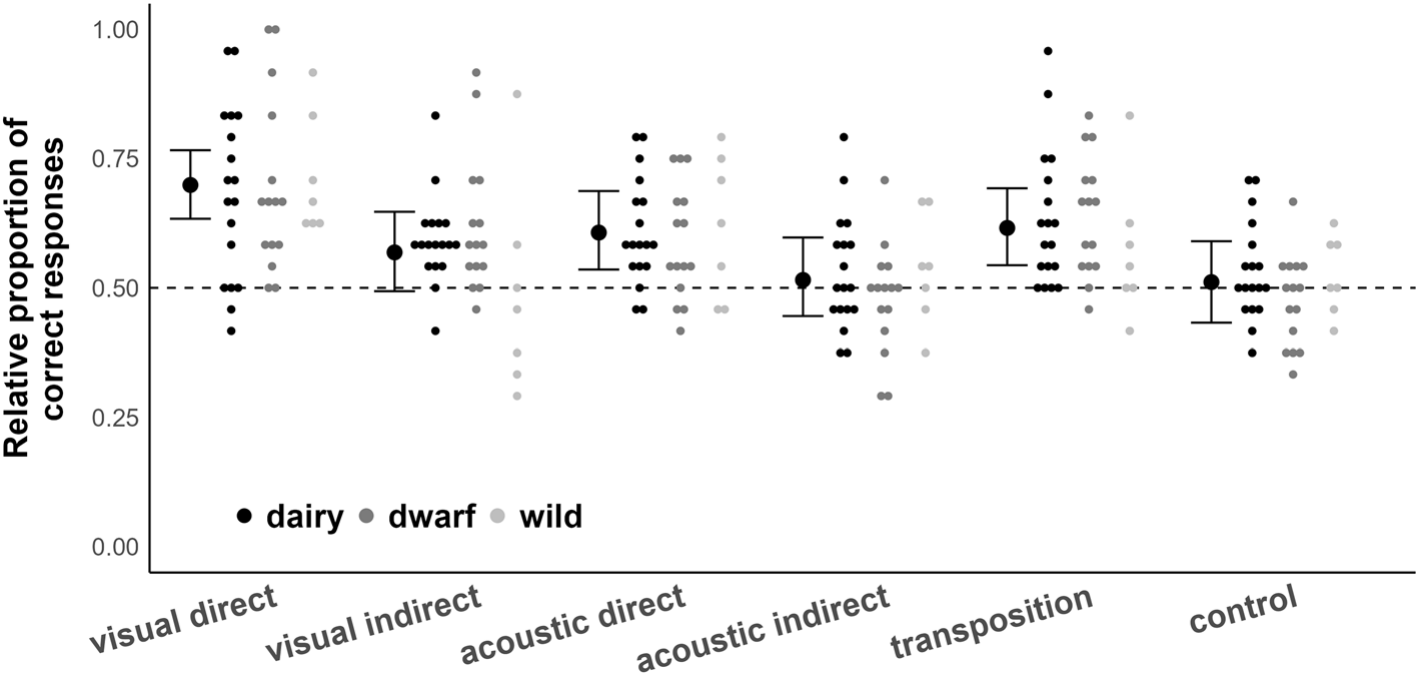
Performance of goats in interpreting physical cues. Small dots represent relative performance of dairy, dwarf and wild goats in the five different physical cue conditions plus control condition. Thick black dots are the model estimates for each condition, and thin black lines and whiskers are the 95% confidence intervals of the maximum model (including the main effects and interactions). Dotted line represents performance at chance level (50%).

**Figure 3:**
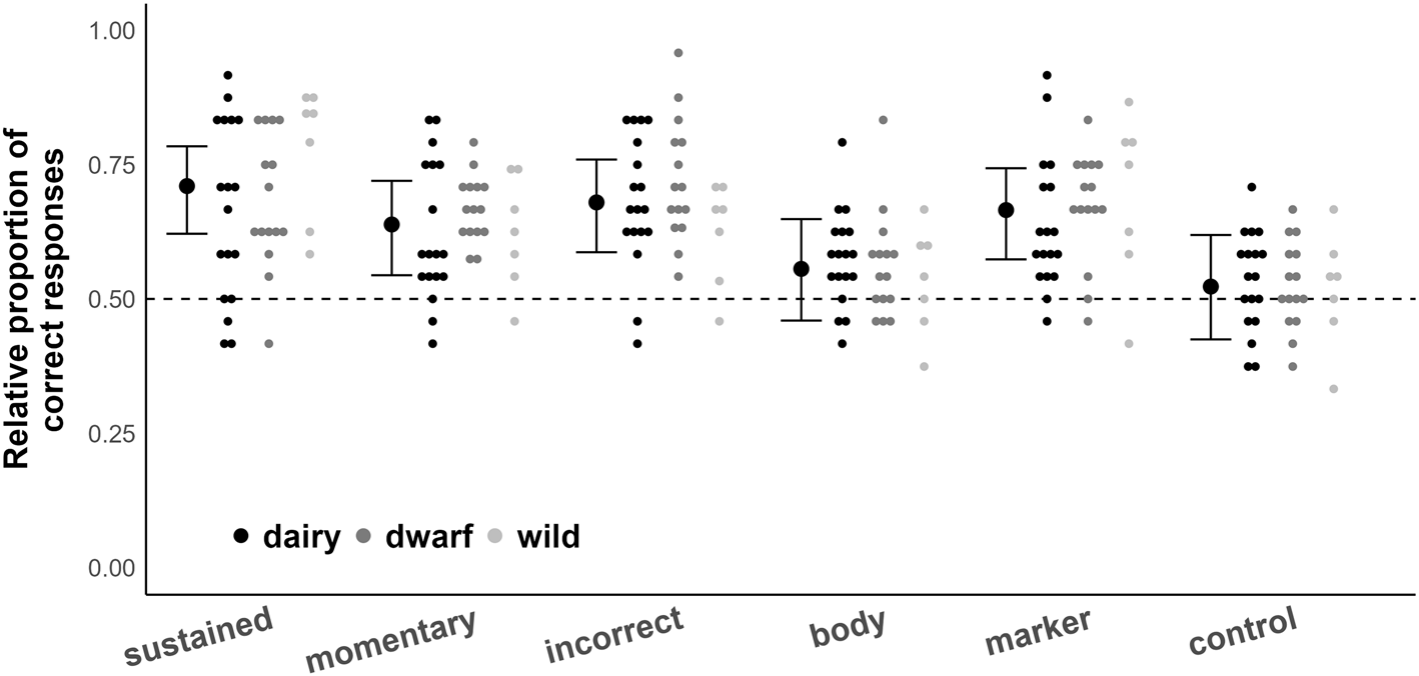
Performance of goats in interpreting social cues. Small dots represent relative performance of dairy, dwarf and wild goats in the five different social cue conditions plus control condition. Thick black dots are the model estimates for each condition, and thin black lines and whiskers are the 95% confidence intervals of the maximum model (including the main effects and interactions). Dotted line represents performance at chance level (50%).

We detected no statistical difference regarding interaction effects between ‘Condition’ and ‘Group’ in neither model (physical: P = 0.12, social: P = 0.15). Our data do not support a statistical difference between the three groups in their performance in using physical and social cues (physical cues: P = 0.74; social cues: P = 0.79, see Figs. 2 and 3).

Goats, independent of group, differed in their interpretation of physical cues (P = 0.0012): they were able to use all physical cues, except the indirect visual and the indirect acoustic information, to locate the reward (model estimate [low.CI; up.CI]: visual direct: 0.70 [0.63; 0.76]; visual indirect: 0.57 [0.49; 0.65]; acoustic direct: 0.61 [0.53; 0.68]; acoustic indirect: 0.52 [0.43; 0.60]; transposition: 0.62 [0.54; 0.69]). A deviation of the performance in the control condition from random chance level was not detected (0.51 [0.43; 0.59]).

Goats, independent of group, also differed in their interpretation of social cues (P = 0.0011): they were able to use all social cues, except E’s body orientation, to locate the reward (model estimate [low.CI; up.CI]: sustained: 0.71 [0.63; 0.79]; momentary: 0.64 [0.54; 0.72]; incorrect: 0.68 [0.60; 0.76]; body: 0.56 [0.46; 0.65]; marker: 0.67 [0.58; 0.75]). A deviation of the performance in the control condition from random chance level was not detected (0.52 [0.43; 0.62]).

## Discussion

We investigated whether domestication and a specific breeding objective for high production have affected the ability of goats to interpret physical and social cues. Contrary to our initial hypotheses, we did not find any difference in test performance between the three groups for both, physical and social, stimuli—all three groups performed similarly well in the test battery. These findings are particularly surprising given the extensive background literature on dog-wolf comparisons and on dog breeds, specifically regarding the social domain^14,51^. Our results highlight that the impact of domestication and the breeding objective on the cognitive capacities of domestic animals may require a differential explanation when it comes to animals domesticated and bred for production traits rather than companionship^7,30^.

Wild and domestic goat groups did not differ in their performance in the test battery, neither in their use of physical nor social cues. Contrary to recent research in dogs^17^, but see^24^, wild and domestic goats did not differ in their problem-solving performance—here: in their ability to use physical cues to infer the location of a reward. In turn, the findings in the social domain are particularly surprising given much of the background literature on dog-wolf comparisons in this area indicating that dogs outperform wolves in their ability to interpret human-given cues^7,52^, but see^17^. However, these dog-wolf comparisons have to be approached with caution, as recent research has proposed that wolves’ baseline capacities for social attention and cooperation have been underestimated (the so-called Canine Cooperation Hypothesis^53^). In contrast to wolves, goats are not recognized for their cooperative behaviour during foraging. Hence, the overall performance of goats in the social tasks, especially among wild goats, raises intriguing questions about the factors underlying these abilities. This is particularly surprising as many wild animals usually perform worse in their use of social cues from humans compared to domestic individuals^29,52^, but see^54,55^. Other comparative approaches to assess domestication effects on cognitive performance in non-companion domestic animals are scarce: in some cases, wild counterparts simply no longer exist, as is the case with cattle. In other cases, an abundance of studies has explored the cognitive abilities of the domesticated form of a species, such as horses^56,57^, but a direct comparison with closely related wild counterparts, such as the Przewalski horse, has yet to be undertaken. One noteworthy exception is the study conducted by Albiach-Serrano et al.^30^, which systematically assessed cognition in domestic pigs and compared it with the performance of wild boar. Intriguingly, wild boar also exhibited the ability to interpret human pointing gestures. The good performance in the socio-cognitive tasks in our wild goats further questions a general domestication effect on cognition in non-companion domestic animals.

We also did not find differences between dwarf and dairy goats in their performance in the physical and social realm. Breeding for high performance can lead to decreases in extensive foraging and social behaviours^58^, but might also decrease behavioural flexibility in farm animals^44^, but see^59^. The difference in resource allocation apparently did not affect domestic goats’ performance in using physical cues to a biological relevant degree. Given that breeds selected for high production are also often indirectly selected for lower emotional reactivity (i.e., to avoid injuries of handlers and stress during handling in animals), we would have expected that both selection lines would differ, at least, in their use of social cues. Indeed, in a follow-up experiment using the total sample of goats available at the research stations, we did see a lower reactivity of dairy goats compared to dwarf goats in a novel human test where subjects where individually confronted with an unfamiliar person for a brief period of time^60^, although ontogenetic differences have also to be accounted for. Emotional reactivity alone might thus not be sufficient to impact test performance in our population of goats.

The performances of goats in the different test conditions confirms results from previous goat cognition research and extends our knowledge on their ability to use, and potentially comprehend, physical and social information. While previous research has shown that goats are indeed able to follow a variety of pointing gestures^33,37,61^ and follow the trajectory of hidden objects^34^, our results show that goats also rely on a marker as social cue to locate food, and are able to use direct acoustic information, i.e., a rattling noise, to locate the reward. Variation in performance in all test conditions appears to be suitable for subsequent experimental approaches to identify additional individual parameters in goats that can explain this variability and whether individual cognitive performance itself is stable over time and/or context^62^. The lack of differences in group performances is also specifically surprising as we used at least one test condition for each of the test batteries that would likely lead to a stark drop in performance if subjects would follow other enhancement cues (physical cues: if in the transposition condition subjects developed a spatial strategy towards the location where the reward was last seen, we would expect a performance below 50% success; social cues: if subjects would simply make their choice due to local/stimulus enhancement effects (e.g. the body of the experimenter), we would expect to see a performance below 50% success in the condition where a pointing gesture was administered from a human positioned behind the incorrect location). Visual inspection of Figs. 2 and 3 does, however, indicate that none of the groups showed stark differences in variation in these two conditions.

The number of subjects, and in particular the number of wild goats tested, can potentially limit our conclusions. We opted to opportunistically test all subjects from the wild goat population that were confident in interacting with the sliding board and the experimenter. This self-selection of focal subjects skewed our sample towards individuals that were, at the age of testing, older compared to the subjects from the domestic groups. As frequent and positive human contact appears to be needed to develop good point following skills^63^, this age difference might have biased their performance (see^30^ for wild boar). From our own observations at the site, we neither observed physical interactions between wild goats and visitors, or wild goats and handlers, prior to habituation to the test set-up. We also did not observe wild goats in the proximity of the handlers when the latter were entering the enclosure. Any effect of age, or experience of prior positive interactions with humans, might thus be considered relatively weak. An additional limitation of this self-selection might have been that we could only selectively test those subjects that had an inert predisposition to engage with humans in general. Unfortunately, our current set-up did not offer the opportunity to falsify this hypothesis as we were unable to test those individuals that continued to show high stress responses in the proximity of humans. This issue is, indeed, also a problem for many other species being tested, wild or captive (including a self-selection bias for owners in canine research). Interestingly, it was only subjects which would be considered rather juvenile that approached the fence, while full adults either never interacted with the experimenter or quickly lost interest. Another potential shortcoming is the sole use of an object choice task to assess potential differences in cognitive performance. We opted to choose test conditions based on this “cup task” as this would guarantee a somewhat standardized testing of domestic and wild goats. Designs that would rely on isolating subjects from the group (e.g., problem-solving task via a puzzle box) or on humans entering the wild goat enclosure (e.g., assessment of attachment bonds) would not be suitable as wild goats reacted very aversive against isolation and close human contact.

## Conclusions

Contrary to our predictions and most of the background literature on other species, we did not detect differences in the performances between wild, dwarf and dairy goats in their use of physical and social cues. Thus, artificial selection by humans, either over the course of domestication or via subsequent selection for production traits, might have a much smaller impact on cognitive capacities than previously thought^4,6^, but see^64,65^.

### Supplementary Information

Supplementary Information 1.

Supplementary Information 2.

Supplementary Information 3.

Supplementary Video 1.

Supplementary Information 4.

Supplementary Information 5.

## Data Availability

Code and raw data are available at the Electronic Supplementary Material (ESM) and here: https://osf.io/fcyg3/.
