# Supplementary material for: Domestication and breeding objective did not shape the interpretation of physical and social cues in goats (Capra hircus)
Source: Sci Rep. 2023 Nov 4;13:19098. doi: 10.1038/s41598-023-46373-9 (PMC10625633; doi:10.1038/s41598-023-46373-9)
Supplement: Supplementary file 1 — Supplementary Information 1. [file 41598_2023_46373_MOESM1_ESM.docx]

**ELECTONIC SUPPLEMENTARY MATERIAL**

**Supplementary table S1** Information on individual study subjects

**Supplementary text S2** Detailed description of the habituation and training procedure for wild and domestic goats

**Supplementary video S3** Illustration of a test trial using the sustained pointing gesture with all three groups of goats (wild goat, dwarf goat, dairy goats)

**Supplementary text S4** R script of data analysis

**Supplementary table S5** Raw data file
