# Supplementary material for: Domestication and breeding objective did not shape the interpretation of physical and social cues in goats (Capra hircus)
Source: Sci Rep. 2023 Nov 4;13:19098. doi: 10.1038/s41598-023-46373-9 (PMC10625633; doi:10.1038/s41598-023-46373-9)
Supplement: Supplementary file 3 — Supplementary Information 3. [file 41598_2023_46373_MOESM3_ESM.docx]

Supplementary Information:

**Domestication and breeding objective did not shape the interpretation of physical and social cues in goats (*Capra hircus*)**

Christian Nawroth, Katrina Wiesmann, Peter Schlup, Nina Keil, Jan Langbein

**Description of habituation to isolation, shaping and training prior to the cognitive test procedures in domestic goats**

All domestic goats were habituated to the test arena. At first the whole group of a pen (2 d; 20 min per day), then in groups of two (4–6 d), and finally individually (7–13 d). The habituation continued until each animal confidently took a reward (a piece of uncooked pasta) from the sliding board. In a following ‘shaping’ phase (10 trials per session), goats were trained to indicate a choice. A small bowl (2 cm high, Ø 14 cm) with a reward (dry pasta) was placed in the middle of the sliding board (4 trials). As soon as the animal stuck its nose through the middle of the grate, it received the reward from the experimenter. For more details on the habituation protocol see Nawroth et al. 2022. In the next step, the reward was covered with a cup (light brown, 10.5 cm high, Ø 12 cm) before the animal could make a choice (6 trials). The goal of the shaping phase was achieved, if at the end of the shaping phase, the animals showed no signs of stress during the manipulation of and feeding from the bowl. This phase was followed by further training sessions of 10 trials, where 2 cups with bowls were positioned on the left and right side of the sliding board (30 cm distance). The experimenter baited, visible to the goat, one of the bowls, and then covered both bowls with a cup. The goat received the reward only if it chose the baited cup. The rewarded pot cup presented in a pseudo-randomised manner on each side. Subjects were considered to have completed training when they achieved at least eight out of ten correct choices in two consecutive sessions (binomial test; P = 0.012). Dwarf goats needed 2.94 ± 0.26 (mean ± SEM) sessions to reach the criterion (Ettenhausen 2.50 ± 0.27 sessions; Dummerstorf: 3.33 ± 0.41 sessions), while dairy goats needed 2.78 ± 0.21 sessions to reach the criterion (Ettenhausen 2.78 ± 0.32 sessions; Dummerstorf: 2.78 ± 0.28 sessions).

**Description of habituation to isolation, shaping and training to the cognitive test procedures in wild goats**

The equipment (bowls, cups, sliding board) used was similar to the one used for domestic goats. Due to the opportunistic nature of the testing (individual subjects had to be tested in a group setting, with possible interferences by other subjects), no systematic group and dyadic habituation could take place. Trials administered per day for shaping and training could vary, and sessions had to be split over several days. Criteria for shaping and training remained the same as for domestic goats. Wild goats needed 3.57 ± 1.02 (mean ± SEM) sessions to reach the criterion. Five other subjects frequently explored the test arena but never showed motivation to obtain food rewards from the experimenter (or the experimental apparatus).
