# Supplementary material for: Domestication and breeding objective did not shape the interpretation of physical and social cues in goats (Capra hircus)
Source: Sci Rep. 2023 Nov 4;13:19098. doi: 10.1038/s41598-023-46373-9 (PMC10625633; doi:10.1038/s41598-023-46373-9)
Supplement: Supplementary file 5 — Supplementary Information 4. [file 41598_2023_46373_MOESM5_ESM.docx]

## Paper: Domestication and breeding objective did not shape the ##

## interpretation of physical and social cues in goats ##

## ##

## Authors: C. Nawroth, K. Wiesmann, P. Schlupp, N. Keil, J. Langbein ##

## ##

## Code Author: CN ##

## ##

## Version: 1.0 ##

## ##

## Software: R 4.1.1 ##

### Load packages ----------------------------------------------------------------

library(blme)

library(tidyverse)

library(pbkrtest)

library(DHARMa)

library(boot)

library(see)

### Options ---------------------------------------------------------------------

options(scipen=999) # force R to avoid all scientific notation

### Themes -----------------------------------------------------------------------

my_theme = theme_modern() +

theme(plot.title = element_text(face="bold", size=16),

axis.title = element_text(face="bold", size=16),

axis.text.x = element_text(face="bold", size=16, angle=15, hjust=1),

axis.text.y = element_text(size=12),

strip.text = element_text(size = 12),

panel.grid.minor = element_blank(),

axis.title.x = element_blank(),

legend.position = c(0.2, 0.1),

legend.direction = "horizontal",

legend.margin = margin(0.2, 0.2, 0.2, 0.2, "cm"),

legend.title=element_blank(),

legend.text = element_text(face="bold", size=16))

### Import Data ------------------------------------------------------------------

goats.df <- read.delim("raw_data.csv", header = TRUE, sep= ';')

physical.df <- subset(goats.df, type == "physical",

select=c(breed:correct))

str(physical.df)

summary(physical.df)

physical.df$ID<-as.factor(physical.df$ID)

physical.df$breed<-as.factor(physical.df$breed)

physical.df$condition<-as.factor(physical.df$condition)

physical.df$session<-factor(physical.df$session, order=TRUE)

physical.df$trial<-factor(physical.df$trial, order=TRUE)

physical.df$experimenter<-as.factor(physical.df$experimenter)

physical.df$location<-as.factor(physical.df$location)

physical.df$pen<-as.factor(physical.df$pen)

physical.df$baited_side<-as.factor(physical.df$baited_side)

physical.df$correct<-as.numeric(physical.df$correct)

social.df <- subset(goats.df, type == "social",

select=c(breed:correct))

str(social.df)

summary(social.df)

social.df$ID<-as.factor(social.df$ID)

social.df$breed<-as.factor(social.df$breed)

social.df$condition<-as.factor(social.df$condition)

social.df$session<-factor(social.df$session, order=TRUE)

social.df$trial<-factor(social.df$trial, order=TRUE)

social.df$experimenter<-as.factor(social.df$experimenter)

social.df$location<-as.factor(social.df$location)

social.df$pen<-as.factor(social.df$pen)

social.df$baited_side<-as.factor(social.df$baited_side)

social.df$correct<-as.numeric(social.df$correct)

### Model Physical Cues ------------------------------------------------------------------

contrasts (physical.df [, 'condition']) <- contr.sum (6)

contrasts (physical.df [, 'breed']) <- contr.sum (3)

contr = glmerControl(optimizer = "bobyqa",

optCtrl = list(maxfun = 10000000),

calc.derivs = FALSE)

physical.df <- cbind(physical.df, model.matrix (~ condition + breed, physical.df) [,-1])

physical.bglmer.full <- bglmer(correct ~ 1 + condition1 + condition2 + condition3 + condition4 + condition5

+ breed1 + breed2

+ breed1 : condition1 + breed1 : condition2 + breed1 : condition3 + breed1 : condition4 + breed1 : condition5

+ breed2 : condition1 + breed2 : condition2 + breed2 : condition3 + breed2 : condition4 + breed2 : condition5

+ (1|experimenter) + (1|location/pen/ID), family = binomial, data = physical.df, na.action=na.exclude, control=contr, REML = FALSE)

physical.bglmer.null <- bglmer(correct ~ 1 +

+ (1|experimenter) + (1|location/pen/ID), family = binomial, data = physical.df, na.action=na.exclude, control=contr, REML = FALSE)

physical.bglmer.int <- bglmer(correct ~ 1 + condition1 + condition2 + condition3 + condition4 + condition5

+ breed1 + breed2

+ (1|experimenter) + (1|location/pen/ID), family = binomial, data = physical.df, na.action=na.exclude, control=contr, REML = FALSE)

physical.bglmer.breed <- bglmer(correct ~ 1 + condition1 + condition2 + condition3 + condition4 + condition5

+ breed1 : condition1 + breed1 : condition2 + breed1 : condition3 + breed1 : condition4 + breed1 : condition5

+ breed2 : condition1 + breed2 : condition2 + breed2 : condition3 + breed2 : condition4 + breed2 : condition5

+ (1|experimenter) + (1|location/pen/ID), family = binomial, data = physical.df, na.action=na.exclude, control=contr, REML = FALSE)

physical.bglmer.cond <- bglmer(correct ~ 1

+ breed1 + breed2

+ breed1 : condition1 + breed1 : condition2 + breed1 : condition3 + breed1 : condition4 + breed1 : condition5

+ breed2 : condition1 + breed2 : condition2 + breed2 : condition3 + breed2 : condition4 + breed2 : condition5

+ (1|experimenter) + (1|location/pen/ID), family = binomial, data = physical.df, na.action=na.exclude, control=contr, REML = FALSE)

# Bootstrapping

set.seed(1000)

physical_bs_null <- PBmodcomp(physical.bglmer.full, physical.bglmer.null)

physical_bs_int <- PBmodcomp(physical.bglmer.full, physical.bglmer.int)

physical_bs_cond <- PBmodcomp(physical.bglmer.full, physical.bglmer.cond)

physical_bs_breed <- PBmodcomp(physical.bglmer.full, physical.bglmer.breed)

summary(physical_bs_null)

summary(physical_bs_int)

summary(physical_bs_cond)

summary(physical_bs_breed)

# Covariance by random factors

VarCorr(physical.bglmer.full)

# Model assumptions

sim.res <- simulateResiduals (physical.bglmer.full)

plot (sim.res)

testResiduals(sim.res)

plotResiduals (sim.res, physical.df [, 'condition'])

plotResiduals (sim.res, physical.df [, 'breed'])

# Calculating CIs

extract.ci <- function (x) {

out <- data.frame (numeric (0),

numeric (0),

numeric (0))

for (i in 1:length (x [['t0']])) {

out <- rbind (out, c (x [['t0']] [i],

boot.ci (x, index= i,

type= 'perc') [['percent']] [, 4:5]))

}

names (out) <- c ('estim', 'lo.ci', 'up.ci')

out

}

physical.bglmer.full.estim.dat <- data.frame(breed1= rep (0, 6),

breed2= rep (0, 6),

condition1= c (1, 0, 0, 0, 0, -1),

condition2= c (0, 1, 0, 0, 0, -1),

condition3= c (0, 0, 1, 0, 0, -1),

condition4= c (0, 0, 0, 1, 0, -1),

condition5= c (0, 0, 0, 0, 1, -1))

physical.bglmer.full.estim.Mod <- function (x) predict (x, physical.bglmer.full.estim.dat, re.form= NA)

physical.bglmer.full.estim.raw <- bootMer (physical.bglmer.full, physical.bglmer.full.estim.Mod, nsim= 10, .progress= 'win')

physical.bglmer.full.estim.val <- apply (extract.ci (physical.bglmer.full.estim.raw),

2, inv.logit)

physical.bglmer.full.estim.val [, 'estim']

physical.bglmer.full.estim.val [, 'lo.ci']

physical.bglmer.full.estim.val [, 'up.ci']

physical.bglmer.full.CIs <- data.frame(physical.bglmer.full.estim.val)

cond <- c(1,2,3,4,5,6)

physical.bglmer.full.CIs <- cbind(physical.bglmer.full.CIs, condition = cond)

### Figure Physical Cues ------------------------------------------------------------------

phys_mean <- physical.df %>%

group_by(breed, ID, condition) %>%

summarize(mean_correct = mean(correct), n = n())

phys_mean$ID<-as.factor(phys_mean$ID)

phys_mean$breed<-as.factor(phys_mean$breed)

phys_mean$condition<-as.factor(phys_mean$condition)

physical_label <- c("visual direct", "visual indirect", "acoustic direct", "acoustic indirect", "transposition", "control")

fig_physical = ggplot() +

scale_x_discrete(labels=physical_label) +

geom_hline(aes(yintercept=0.5), linetype="dashed") +

ylim(0, 1) +

ylab("Relative proportion of

correct responses") +

geom_dotplot(data=phys_mean, aes(x=condition, y=mean_correct, fill = factor(breed), color = factor(breed)), binaxis='y', stackdir='center',

dotsize = 0.4, position = position_dodge(0.6)) +

geom_point(data=physical.bglmer.full.CIs, col="black", size=3,

aes(x=as.numeric(condition)+-0.4, y=estim)) +

geom_errorbar(data=physical.bglmer.full.CIs, col="black", width=0.2,

aes(x=as.numeric(condition)+-0.4,

ymin=lo.ci, ymax=up.ci))+

my_theme +

scale_fill_manual(values = c("#343434", "#6E6E6E", "#B0B0B0"))+

scale_color_manual(values = c("#343434", "#6E6E6E", "#B0B0B0"))

fig_physical

ggsave("physical.tiff", width = 25, height = 12, units = "cm")

### Model Social Cues ------------------------------------------------------------------

contrasts (social.df [, 'condition']) <- contr.sum (6)

contrasts (social.df [, 'breed']) <- contr.sum (3)

contr = glmerControl(optimizer = "bobyqa",

optCtrl = list(maxfun = 10000000),

calc.derivs = FALSE)

social.df <- cbind(social.df, model.matrix (~ condition + breed, social.df) [,-1])

social.bglmer.full <- bglmer(correct ~ 1 + condition1 + condition2 + condition3 + condition4 + condition5

+ breed1 + breed2

+ breed1 : condition1 + breed1 : condition2 + breed1 : condition3 + breed1 : condition4 + breed1 : condition5

+ breed2 : condition1 + breed2 : condition2 + breed2 : condition3 + breed2 : condition4 + breed2 : condition5

+ (1|experimenter) + (1|location/pen/ID), family = binomial, data = social.df, na.action=na.exclude, control=contr, REML = FALSE)

social.bglmer.null <- bglmer(correct ~ 1 +

+ (1|experimenter) + (1|location/pen/ID), family = binomial, data = social.df, na.action=na.exclude, control=contr, REML = FALSE)

social.bglmer.int <- bglmer(correct ~ 1 + condition1 + condition2 + condition3 + condition4 + condition5

+ breed1 + breed2

+ (1|experimenter) + (1|location/pen/ID), family = binomial, data = social.df, na.action=na.exclude, control=contr, REML = FALSE)

social.bglmer.breed <- bglmer(correct ~ 1 + condition1 + condition2 + condition3 + condition4 + condition5

+ breed1 : condition1 + breed1 : condition2 + breed1 : condition3 + breed1 : condition4 + breed1 : condition5

+ breed2 : condition1 + breed2 : condition2 + breed2 : condition3 + breed2 : condition4 + breed2 : condition5

+ (1|experimenter) + (1|location/pen/ID), family = binomial, data = social.df, na.action=na.exclude, control=contr, REML = FALSE)

social.bglmer.cond <- bglmer(correct ~ 1

+ breed1 + breed2

+ breed1 : condition1 + breed1 : condition2 + breed1 : condition3 + breed1 : condition4 + breed1 : condition5

+ breed2 : condition1 + breed2 : condition2 + breed2 : condition3 + breed2 : condition4 + breed2 : condition5

+ (1|experimenter) + (1|location/pen/ID), family = binomial, data = social.df, na.action=na.exclude, control=contr, REML = FALSE)

# Bootstrapping

set.seed(1000)

social_bs_null <- PBmodcomp(social.bglmer.full, social.bglmer.null)

social_bs_int <- PBmodcomp(social.bglmer.full, social.bglmer.int)

social_bs_cond <- PBmodcomp(social.bglmer.full, social.bglmer.cond)

social_bs_breed <- PBmodcomp(social.bglmer.full, social.bglmer.breed)

summary(social_bs_null)

summary(social_bs_int)

summary(social_bs_cond)

summary(social_bs_breed)

# Covariance by random factors

VarCorr(social.bglmer.full)

# Model assumptions

sim.res <- simulateResiduals (social.bglmer.full)

plot (sim.res)

testResiduals(sim.res)

plotResiduals (sim.res, social.df [, 'condition'])

plotResiduals (sim.res, social.df [, 'breed'])

# Calculating CIs

extract.ci <- function (x) { ## Hilfsfunktion

out <- data.frame (numeric (0),

numeric (0),

numeric (0))

for (i in 1:length (x [['t0']])) {

out <- rbind (out, c (x [['t0']] [i],

boot.ci (x, index= i,

type= 'perc') [['percent']] [, 4:5]))

}

names (out) <- c ('estim', 'lo.ci', 'up.ci')

out

}

social.bglmer.full.estim.dat <- data.frame(breed1= rep (0, 6),

breed2= rep (0, 6),

condition1= c (1, 0, 0, 0, 0, -1),

condition2= c (0, 1, 0, 0, 0, -1),

condition3= c (0, 0, 1, 0, 0, -1),

condition4= c (0, 0, 0, 1, 0, -1),

condition5= c (0, 0, 0, 0, 1, -1))

social.bglmer.full.estim.Mod <- function (x) predict (x, social.bglmer.full.estim.dat, re.form= NA)

social.bglmer.full.estim.raw <- bootMer (social.bglmer.full, social.bglmer.full.estim.Mod, nsim= 1000, .progress= 'win')

social.bglmer.full.estim.val <- apply (extract.ci (social.bglmer.full.estim.raw),

2, inv.logit)

social.bglmer.full.estim.val [, 'estim']

social.bglmer.full.estim.val [, 'lo.ci']

social.bglmer.full.estim.val [, 'up.ci']

social.bglmer.full.CIs <- data.frame(social.bglmer.full.estim.val)

cond <- c(1,2,3,4,5,6)

social.bglmer.full.CIs <- cbind(social.bglmer.full.CIs, condition = cond)

### Figure Social Cues ------------------------------------------------------------------

soc_mean <- social.df %>%

group_by(breed, ID, condition) %>%

summarize(mean_correct = mean(correct), n = n())

soc_mean$ID<-as.factor(soc_mean$ID)

soc_mean$breed<-as.factor(soc_mean$breed)

soc_mean$condition<-as.factor(soc_mean$condition)

social_label <- c("sustained", "momentary", "incorrect", "body", "marker", "control")

fig_social = ggplot() +

scale_x_discrete(labels=social_label) +

geom_hline(aes(yintercept=0.5), linetype="dashed") +

ylim(0, 1) +

ylab("Relative proportion of

correct responses") +

geom_dotplot(data=soc_mean, aes(x=condition, y=mean_correct, fill = factor(breed), color = factor(breed)), binaxis='y', stackdir='center',

dotsize = 0.4, position = position_dodge(0.6)) +

geom_point(data=social.bglmer.full.CIs, col="black", size=3,

aes(x=as.numeric(condition)+-0.4, y=estim)) +

geom_errorbar(data=social.bglmer.full.CIs, col="black", width=0.2,

aes(x=as.numeric(condition)+-0.4,

ymin=lo.ci, ymax=up.ci))+

my_theme +

scale_fill_manual(values = c("#343434", "#6E6E6E", "#B0B0B0"))+

scale_color_manual(values = c("#343434", "#6E6E6E", "#B0B0B0"))

fig_social

ggsave("social.tiff", width = 25, height = 12, units = "cm")
